# Continuous Structural Displacement Monitoring Using Accelerometer, Vision, and Infrared (IR) Cameras

**DOI:** 10.3390/s23115241

**Published:** 2023-05-31

**Authors:** Jaemook Choi, Zhanxiong Ma, Kiyoung Kim, Hoon Sohn

**Affiliations:** Department of Civil and Environmental Engineering, Korea Advanced Institute of Science and Technology, Daejeon 34141, Republic of Korea; cjmook@kaist.ac.kr (J.C.); mazhanxiong@kaist.ac.kr (Z.M.); kiyoungkim@kaist.ac.kr (K.K.)

**Keywords:** displacement estimation, infrared camera, vision camera, accelerometer, multirate adaptive Kalman filter, continuous monitoring

## Abstract

With the rapid development of computer vision, vision cameras have been used as noncontact sensors for structural displacement measurements. However, vision-based techniques are limited to short-term displacement measurements because of their degraded performance under varying illumination and inability to operate at night. To overcome these limitations, this study developed a continuous structural displacement estimation technique by combining measurements from an accelerometer with vision and infrared (IR) cameras collocated at the displacement estimation point of a target structure. The proposed technique enables continuous displacement estimation for both day and night, automatic optimization of the temperature range of an infrared camera to ensure a region of interest (ROI) with good matching features, and adaptive updating of the reference frame to achieve robust illumination–displacement estimation from vision/IR measurements. The performance of the proposed method was verified through lab-scale tests on a single-story building model. The displacements were estimated with a root-mean-square error of less than 2 mm compared with the laser-based ground truth. In addition, the applicability of the IR camera for displacement estimation under field conditions was validated using a pedestrian bridge test. The proposed technique eliminates the need for a stationary sensor installation location by the on-site installation of sensors and is therefore attractive for long-term continuous monitoring. However, it only estimates displacement at the sensor installation location, and cannot simultaneously estimate multi-point displacements which can be achieved by installing cameras off-site.

## 1. Introduction

Displacement is a critical parameter that indicates the level of deformation or movement of civil infrastructure [1,2]. Measuring displacement helps to identify potential safety hazards and structural issues that could lead to failure or collapse. By monitoring the displacement, engineers can determine whether a structure is still within the acceptable limits of operation or if it requires repair or reinforcement. A linear variable displacement transducer (LVDT) is commonly used for bridge displacement measurement [3]. However, its usage requires the installation of a scaffold beneath the bridge, which may not be feasible for river crossings and overpass bridges where traffic flow interruptions are not permitted. Although real-time kinematic global navigation satellite systems (RTK-GNSS) have been widely applied for the continuous monitoring of structural displacement in large-scale bridges [4] and tall buildings [5], their precision and sampling rate are restricted, making them inadequate for monitoring small- or medium-scale structures. Though satellite-based interferometry techniques are advantageous for full-field displacement measurements for landslides [6] and bridges [7], they are limited to static displacement monitoring. Owing to the limitations of current displacement sensors, accelerometers are commonly used for the continuous long-term monitoring of structures. However, the displacements estimated from acceleration measurements do not include the critical static and pseudo static components of structural displacement [8,9].

In addition to the abovementioned contact-type displacement measurement techniques, a range of noncontact displacement measurement techniques have been developed based on laser Doppler vibrometers (LDV) [10], radar systems [11,12], and vision cameras [13,14]. LDV and radar systems emit laser light and electromagnetic waves, respectively, toward the surface of a structure and subsequently receive reflected signals. These systems can accurately determine the displacement of a structure by measuring the time delay between the emission and reception of a signal. Although both LDV and radar systems can achieve high-precision measurements, their high cost limits their widespread use. On the other hand, vision cameras capture images of the structure and determine structural displacement by tracking changes in the structure position in these images. Although vision cameras are relatively inexpensive, they are sensitive to environmental conditions such as light and weather, and less accurate and efficient than LDV and radar systems. In addition, all sensors should be fixed at a stationary location, which makes them unsuitable for continuous long-term displacement monitoring rather than short-term measurements.

In recent years, combinations of different types of sensors have become increasingly popular for estimating the structural displacements [15,16]. Such a combination can provide complementary information and help improve the accuracy and efficiency of structural displacement estimation. Accelerometers are commonly fused with other types of sensors [17,18,19,20,21]. The authors previously explored the fusion of the vision camera and accelerometer for structural displacement estimation [22]. The vision camera and accelerometer were installed at a target structure, with the accelerometer measuring the structural acceleration at a high sampling rate, whereas the vision camera tracked a fixed target for the surroundings of the structure at a low sampling rate. Because these two sensors are installed at the same location, their data can be easily fused, resulting in highly accurate and highly efficient displacement estimation at a high sampling rate. In addition, the requirement for a stationary location was eliminated by the direct installation of these two sensors on the target structure, making these techniques more appropriate for long-term continuous displacement estimation. Nevertheless, vision cameras are incapable of working at night, which limits the practical application of the proposed technique to long-term continuous structural displacement monitoring.

In this study, a structural displacement estimation technique was developed by fusing accelerometers with vision and infrared (IR) cameras, particularly for long-term continuous displacement monitoring. Three sensors were installed at the displacement estimation point of the target structure, and their initial short-period measurements were first used to automatically estimate the scale factors required for unit conversion and to optimize the temperature range of the selected region of interest (ROI) for the IR camera. The proposed technique then continuously estimates the structural displacements. Specifically, it combines a vision camera and accelerometer to estimate the structural displacement during the day and an IR camera and accelerometer to estimate the structural displacement during the night. In addition, an adaptive reference frame updating algorithm was proposed and applied to enhance the robustness of the proposed technique against variations in illumination in the vision camera and temperature in the IR camera. The main contributions of this study are (1) day and night continuous displacement estimation by fusing the accelerometer, IR, and vision cameras; (2) automated optimization of the ROI temperature range of the IR camera for displacement estimation during the night; and (3) improved robustness of vision-based displacement estimation against variations in illumination in the vision camera and temperature in the IR camera by adaptively updating the reference frame.

The remainder of this paper is organized as follows: The proposed continuous displacement estimation technique is described in Section 2. The performance of the proposed technique was experimentally validated using an indoor single-story building model test and outdoor pedestrian bridge test, as described in Section 3. Lastly, the concluding remarks are presented in Section 4.

## 2. Development of Structural Displacement Estimation Technique by Fusing Accelerometer, Vision, and IR Cameras

**Figure 1:**
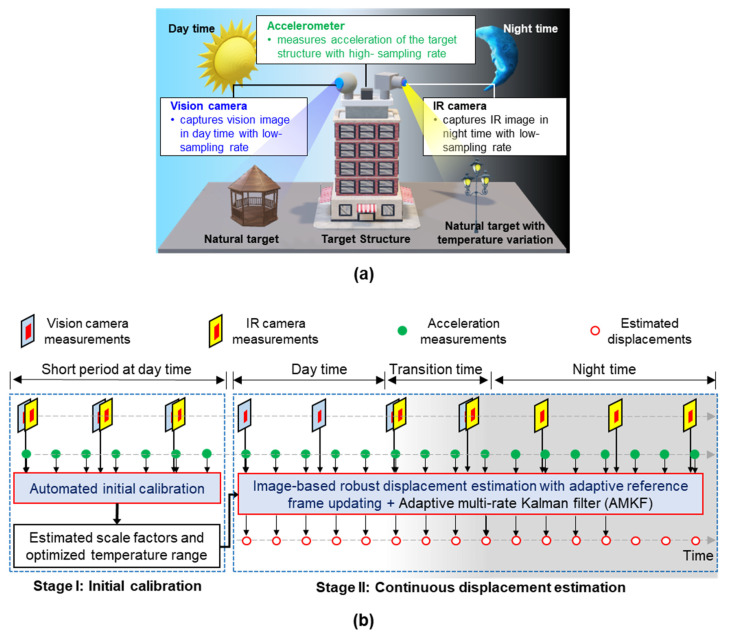
Overview of proposed displacement estimation technique: (**a**) sensor setup and (**b**) overall flowchart for continuous displacement estimation.

This study proposes a continuous displacement estimation method in which acceleration measurements are combined with collected vision and IR images for day and night, respectively. The accelerometer, vision, and IR cameras were mounted at the measurement point of the target structure, and the displacement was estimated, as shown in Figure 1a. The accelerometer measured the acceleration of the target structure at a high sampling rate. However, assuming that the natural targets in the surroundings of the target structures are stationary, the vision camera and IR camera track a natural target with rich features during the day and a natural target with a distinct temperature distribution during the night, both with a low sampling rate. A low sampling displacement was first estimated from the vision/IR images with adaptive reference frame updating, and the image-based displacement was then fused with the high-sampling acceleration using an adaptive multirate Kalman filter. Considering that the movements originally estimated from vision/IR images are in pixel units, the scale factors required to convert these pixel unit movements into structural displacements in a physical unit should be estimated in advance. Additionally, the displacement estimation performance of IR cameras depends on the selection of the temperature range, and it is necessary to optimize the temperature range for better displacement estimation performance. Therefore, the proposed technique is divided into two stages, as shown in Figure 1b: (1) automatic initial calibration for scale factor estimation and temperature range optimization (Section 2.1) and (2) continuous displacement estimation (Section 2.2).

### 2.1. Stage I: Automated Initial Calibration

#### 2.1.1. Scale Factor Estimation for Vision and IR Cameras

**Figure 2:**
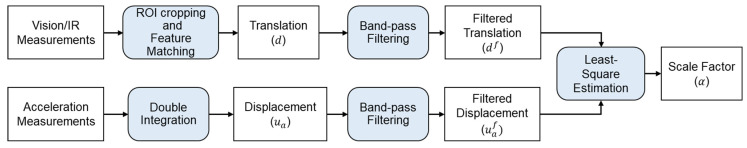
Flowchart of automated scale factor estimation algorithm [22].

In this study, an acceleration-aided algorithm [22] was adopted to automatically estimate the scale factors required for image-based displacement estimation. As shown in Figure 2, translation d was first estimated from the collected short-term vision/IR images after ROI cropping and feature matching. In this study, the speeded-up robust features (SURF) [23] algorithm was used owing to its high accuracy and low computational cost. Subsequently, a bandpass filter was applied to d and the displacement $u_{a}$ was estimated from the double integration of the acceleration measurement. The lower cutoff frequency of the filter was set to be sufficiently high to remove the low-frequency drift in $u_{a}$, and the upper cutoff frequency was set to 1/10 of the vision and IR camera sampling rate [24]. Finally, the scale factor α was estimated as the ratio of filtered translation $d^{f}$ and filtered displacement $u_{a}^{f}$ using a least-squares estimation (LSE) algorithm. Before applying the LSE algorithm, $u_{a}^{f}$ was down-sampled to match the sampling rate with $d^{f}$.

#### 2.1.2. Optimization of the Temperature Range for IR Camera

(a) Necessity of fixing and optimizing the temperature range

**Figure 3:**
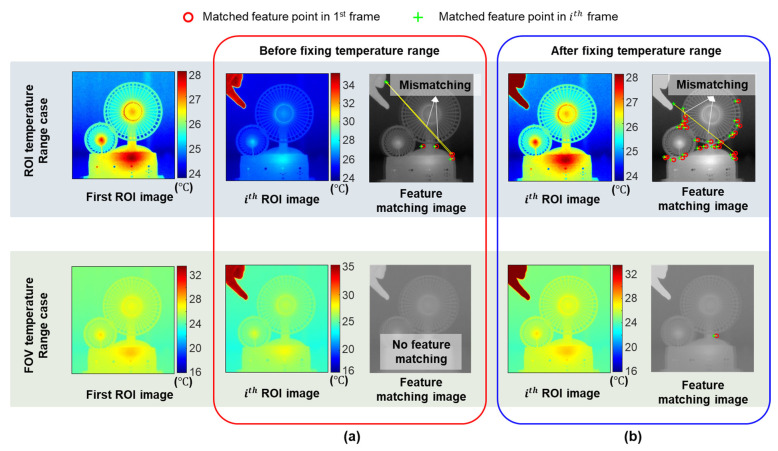
Example of region of interest (ROI) feature-matching results with heat source: (**a**) before fixing temperature range and (**b**) after fixing temperature range.

An IR image is essentially a temperature map, in which different colors represent different temperatures. If the target within the ROI has a stable and distinct temperature distribution, the IR-based displacement can be estimated to be the same as the vision-based displacement by applying a feature-matching algorithm between the reference and current ROIs. However, the difference from vision-based displacement estimation is that only temperature data are contained in an IR image. Therefore, when there is an external extreme heat source in the ROI, the color distribution within the ROI changes, as shown in Figure 3a, causing matching failure or no matching between the reference and the current ROIs. To reduce the above problem, the temperature range of the IR camera was fixed.

Figure 3b shows that the temperature range can be fixed using the maximum and minimum temperatures with the field of view (FOV) ($T_{max}^{F}$ and $T_{min}^{F}$); however, relatively small temperature variations within the ROI cause less distinct features. On the other hand, the temperature range can also be fixed using the maximum and minimum temperatures with the ROI ($T_{max}^{R}$ and $T_{min}^{R}$). Although more distinct features can be detected, they are not stable owing to the temperature measurement noise. Therefore, it is necessary to optimize the temperature range to ensure that sufficient and stable distinct features are available within the ROI.

(b) Working principle of automated optimization of the temperature range

**Figure 4:**
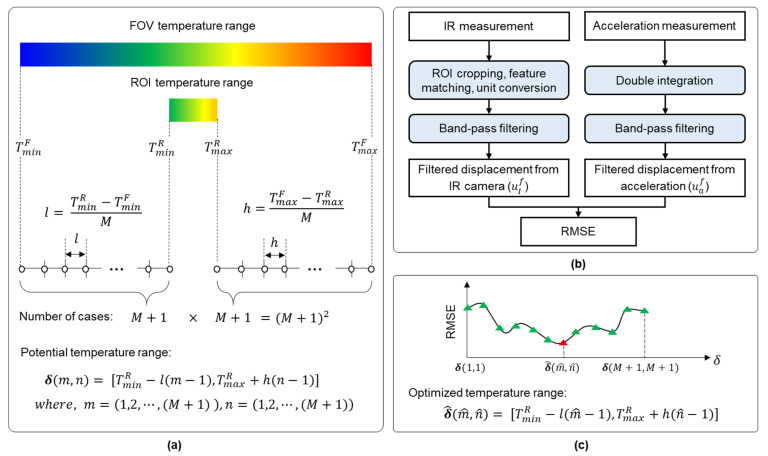
Automated optimization of the temperature range: (**a**) step 1: define potential temperature ranges (δ) with variables m and n, (**b**) step 2: calculate root-mean-square error (RMSE) using initial δ(1,1), and (**c**) step 3: select the optimized temperature range by repeating step 2 with different δ.

The basic idea for temperature range optimization is to calculate the root-mean-square errors (RMSEs) between the acceleration- and IR-based displacements in various temperature ranges. The optimal temperature range was selected when the RMSE was the smallest. The detailed process of the proposed algorithm consists of three steps:

*Step 1*: First, the differences between $T_{min}^{R}$ and $T_{min}^{F}$ and between $T_{max}^{R}$ and $T_{max}^{F}$ were calculated and divided into M equal parts, as follows:

(1) $$l=\frac{T_{min}^{R}-T_{min}^{F}}{M},\;\;h=\frac{T_{max}^{F}-T_{max}^{R}}{M}.$$

where M is a constant value to divide the temperature difference between FOV and ROI into M+1 equal parts. Then, ${\left(M+1\right)}^{2}$ potential temperature ranges (δ) are generated as follows (Figure 4a):

(2) $$\delta \left(m,n\right)=\left[T_{min}^{R}-l\left(m-1\right),T_{max}^{R}+h\left(n-1\right)\right],\;\;m=\left(1,\cdots ,M+1\right),\;n=\left(1,\cdots ,M+1\right).$$

*Step 2*: The displacement was first estimated from the IR measurement using the first temperature range (δ(1,1)) and the estimated scale factor and then bandpass-filtered to obtain the filtered IR-based displacement ($u_{I}^{f}$). The filtered displacement was estimated from the acceleration measurements using double integration and bandpass filtering. Subsequently, the RMSE between the filtered acceleration and IR-based displacements was calculated (Figure 4b).

*Step 3*: Step 2 was repeated for all ${\left(M+1\right)}^{2}$ potential temperature ranges. The temperature range with the smallest RMSE became the optimized temperature range ($\hat{\delta }\left(m,n\right)$). After that, $\hat{\delta }\left(m,n\right)$ was applied to IR-based displacement estimation in Stage II.

### 2.2. Stage II: Continuous Displacement Estimation Using Image-Based Robust Displacement Estimation Algorithm and Adaptive Multirate Kalman Filter (AMKF)

After the initial calibration, the displacement was continuously estimated by fusing the vision/IR-based displacement with asynchronized acceleration measurements using an adaptive multirate Kalman filter (AMKF) [22] developed by our group. The transition between vision and IR cameras is automatically achieved, and the reference frame is adaptively updated to improve the robustness of vision-based displacement estimation against illumination variations and IR-based displacement estimation against temperature variations, which are unavoidable in long-term continuous displacement estimation.

#### 2.2.1. AMKF-Based Fusion of Asynchronous Image and Acceleration Measurement

**Figure 5:**
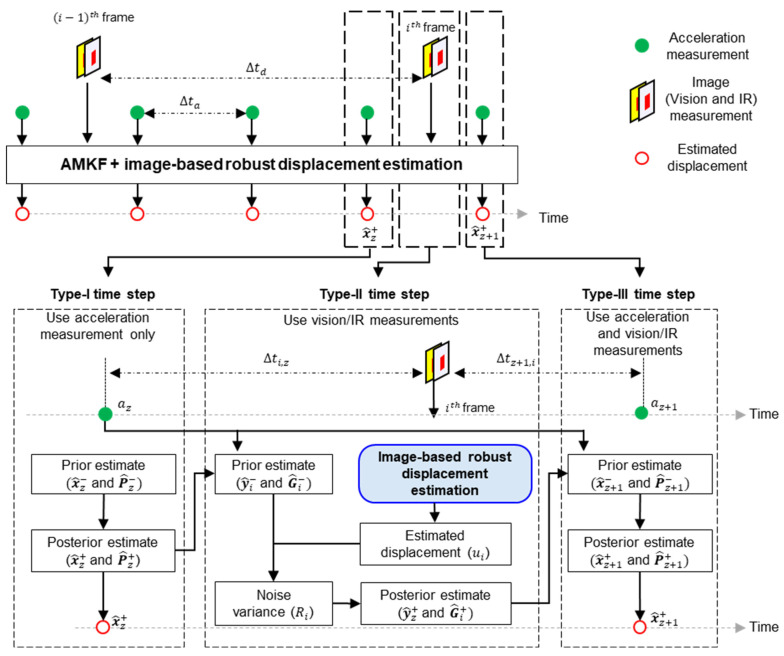
Overview of AMKF-based structural displacement estimation using accelerometer, vision, and infrared (IR) cameras.

Asynchronous accelerations and images were fused using the AMKF, which was formulated for three different time-step types, as shown in Figure 5. In a type-I time step, only acceleration is used, and the state vector (${\hat{x}}_{z}^{+}$), which consists of displacement and velocity, is estimated using the previous time-step state vector (${\hat{x}}_{z-1}^{+}$) and acceleration ($a_{z-1}$).

(3) $${\hat{x}}_{z}^{+}={\hat{x}}_{z}^{-}=A\left(\Delta t_{a}\right)\;{\hat{x}}_{z-1}^{+}+B\left(\Delta t_{a}\right)a_{z-1};\;A\left(\Delta t_{a}\right)=\left[\begin{array}{cc} 1 & \Delta t_{a}\\ 0 & 1 \end{array}\right];B\left(\Delta t_{a}\right)=\left[\begin{array}{c} \frac{\Delta t_{a}^{2}}{2}\\ \Delta t \end{array}\right],$$

where $\Delta t_{a}$ denotes the time interval between the acceleration measurements. Next, the covariance (${\hat{P}}_{z}^{+}$) of ${\hat{x}}_{z}^{+}$ is calculated as follows:

(4) $${\hat{P}}_{z}^{+}={\hat{P}}_{z}^{-}=A\left(\Delta t_{a}\right){\hat{P}}_{z-1}^{+}A^{T}\left(\Delta t_{a}\right)+qB\left(\Delta t_{a}\right)B^{T}\left(\Delta t_{a}\right),$$

where q denotes the noise variance in the acceleration measurements.

In a type-II time step, the prior state (${\hat{y}}_{i}^{-}$) and its covariance (${\hat{G}}_{i}^{-}$) are estimated as follows:

(5) $${\hat{y}}_{i}^{-}=A\left(\Delta t_{i,z}\right)\;{\hat{x}}_{z}^{+}+B\left(\Delta t_{i,z}\right)a_{z},\;{\hat{G}}_{i}^{-}=A\left(\Delta t_{i,z}\right){\hat{P}}_{z}^{+}A^{T}\left(\Delta t_{i,z}\right)+qB\left(\Delta t_{i,z}\right)B^{T}\left(\Delta t_{i,z}\right);\;\Delta t_{i,z}=i\Delta t_{d}-z\Delta t_{a},$$

where $\Delta t_{d}$ denotes the time interval between image measurements. Subsequently, the noise variance ($R_{i}$) in the ith image-based displacement ($u_{i}$) is expressed as follows [25]. The detailed process for estimating $u_{i}$ is described in Section 2.2.2.

(6) $$R_{i}=\beta R_{i-1}+\left(1-\beta \right)\left(\eta_{i}^{2}-H{\hat{G}}_{i}^{-}H^{T}\right),0<\beta <1,\;\;\eta_{i}=u_{i}-H{\hat{y}}_{i}^{-},\;H={\left[\begin{array}{cc} 1 & 0 \end{array}\right]}^{T},$$

where β and η denote the forgetting and innovation factors, respectively. The Kalman gain (K) is calculated as

(7) $$K={\hat{G}}_{i}^{-}H^{T}{\left(H{\hat{G}}_{i}^{-}H^{T}+R_{i}\right)}^{-1}.$$

Finally, ${\hat{y}}_{i}^{-}$ and ${\hat{G}}_{i}^{-}$ are updated in a posterior process using *K* and $u_{i}\;$as follows:

(8) $${\hat{y}}_{i}^{+}=\left(I-KH\right){\hat{y}}_{i}^{-}+Ku_{i},\;{\hat{G}}_{i}^{+}=\left(I-KH\right){\hat{G}}_{i}^{-}.$$

In a type-III time step, the state vector (${\hat{x}}_{z+1}^{+}$) is estimated at the next acceleration time step ($\Delta t_{z+1}$). Details of the AMKF can be found in a study by Ma et al. [22]

(9) $${\hat{x}}_{z+1}^{+}={\hat{x}}_{z+1}^{-}=A\left(\Delta t_{z+1,i}\right){\hat{y}}_{i}^{+}+B\left(\Delta t_{z+1,i}\right)a_{z}\;;\;\Delta t_{z+1,i}=\left(z+1\right)\Delta t_{a}-i\Delta t_{d}\;$$

#### 2.2.2. Image-Based Robust Displacement Estimation with Adaptive Reference Frame Updating

(a) A brief review of the existing algorithm with a fixed reference frame and its limitations

**Figure 6:**
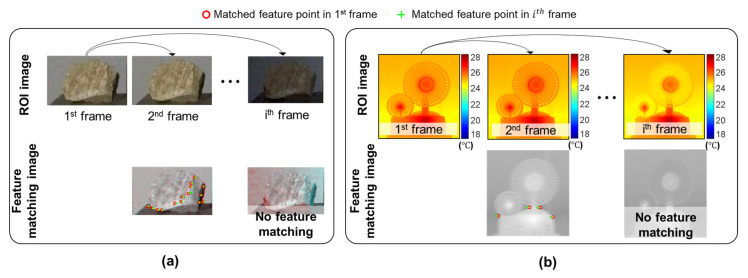
Overview of existing image-based displacement estimation algorithm [22] using a fixed reference frame and its limitation in long-term continuous displacement estimation: (**a**) vision measurement with illumination variation and (**b**) IR measurement with temperature variation.

When estimating displacement from visual measurements using a feature-matching algorithm, existing studies [22] set the first ROI as the reference ROI, and the displacement at each time step was estimated by matching the current and reference ROIs. Therefore, stable illumination conditions are required for a successful displacement estimation. This is not a problem for short-period displacement estimation, which was the focus of these existing studies. However, illumination variation is unavoidable in long-term continuous displacement estimation and may cause insufficient matches, as shown in Figure 6a, making continuous displacement estimation impossible. The IR-based displacement estimation suffers from the same issue. Unavoidable temperature variations may result in an insufficient match, as shown in Figure 6b. Therefore, an algorithm that improves the robustness of vision-based displacement estimation against illumination variations and IR-based displacement estimation against temperature variations is essential for a long-term continuous displacement estimation.

(b) Working principle of adaptive reference frame updating

This study proposes an adaptive reference frame updating algorithm to improve the robustness of vision-based displacement estimation against illumination variations and IR-based displacement estimation against temperature variations. The basic principle of the proposed algorithm is to adaptively update the reference frame when the detected matches are insufficient, and update the reference frame back to the first frame if sufficient matches can be detected.

**Figure 7:**
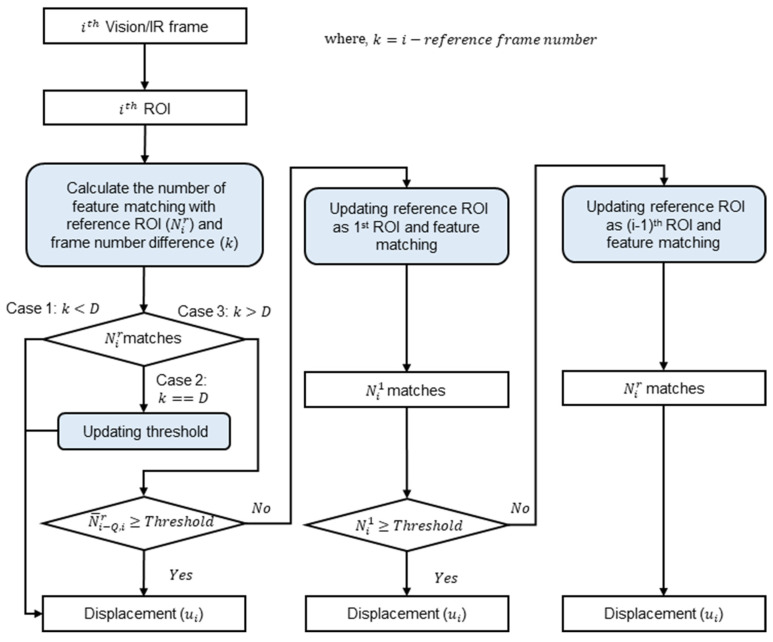
Flowchart of proposed displacement estimation algorithm for estimating displacement from vision/IR images with adaptive reference ROI updating.

Figure 7 shows the working principle of the proposed algorithm. First, after obtaining the ith image from the vision/IR cameras, the ROI was cropped from the FOV. Feature matching was then performed between the ith ROI and the current reference ROI (i.e., the rth ROI) and $N_{i}^{r}$ matches were obtained. Note that the initial reference ROI is the first ROI. Owing to the relatively large variation in the number of matches, even with stable illumination, a moving average filter with an order of (Q+1) was applied to $N_{i}^{r}$ to obtain an average value $\left({\bar{N}}_{i-Q,i}^{r}\right)$.

(10) $${\bar{N}}_{i-Q,i}^{r}=\left(\frac{1}{Q+1}\overset{i}{\underset{j=i-Q}{\sum }}N_{j}^{r}\;\right)$$

If ${\bar{N}}_{i-Q,i}^{r}$ is larger than the threshold ($N_{T}^{r}$), it is not necessary to update the reference frame, and the ith image-based displacement ($u_{i}^{1}$) is calculated as

(11) $$u_{i}^{1}=\alpha d_{i}^{r}+u_{r}^{1},$$

where $d_{i}^{r}$ is the relative translation between the ith and reference ROIs and $u_{r}^{1}$ is the relative displacement between the 1st and reference ROIs. α denotes the scale factor for vision/IR measurements. Note that $N_{T}$ was determined as follows:

(12) $$N_{T}^{r}=\left(\frac{1}{D}\overset{r+D}{\underset{j=r+1}{\sum }}N_{j}^{r}\;\right)-3\sigma \left[N_{j}^{r}|\;j=\left(r+1\right),\;\cdots ,\left(r+D\right)\right].$$

Otherwise, feature matching was performed between the 1st and ith ROIs, and $N_{i}^{1}$ matches were obtained. If $N_{i}^{1}$ is larger than a threshold $\left(N_{T}^{1}\right)$, the reference frame is updated to the first frame, and $u_{i}^{1}$ is estimated as

(13) $$u_{i}^{1}=\alpha d_{i}^{1},$$

where $d_{i}^{1}$ is the relative translation between the ith and 1st ROIs, and $N_{T}^{1}$ is determined as

(14) $$N_{T}^{1}=\left(\frac{1}{D}\overset{D+1}{\underset{j=2}{\sum }}N_{j}^{1}\;\right)-3\sigma \left[N_{j}^{1}|\;j=2,\;\cdots ,\left(D+1\right)\right].$$

Denoting k as the difference in the number of frames between the ith frame and the reference frame, reference frame updating is executed differently in the three cases by comparing the rules of k and D as follows:

*Case 1*: k is less than D. The reference frame changes only within the prior D timesteps. The ith ROI and reference ROI are directly matched, and ith image-based displacement ($u_{i}$) is calculated using Equation (11).

*Case 2*: k = D. Here, the threshold value is calculated by applying Equation (12) using D frames sequentially after the reference frame. The subsequent process was the same as that for Case 1.

*Case 3*: k > D. If ${\bar{N}}_{i-D,i}^{r}$ is larger than the threshold, $u_{i}$ can be estimated using (11). However, if ${\bar{N}}_{i-D,i}^{r}$ is smaller, then the reference frame is updated to the first frame. The number of matches ($N_{i}^{1}$) compared to the first frame was compared to the threshold value ($N_{T}^{1}$). If $N_{i}^{1}$ was larger than $N_{T}^{1}$, $u_{i}$ was calculated using Equation (13); however, if $N_{i}^{1}$ was less than $N_{T}^{1}$, the reference frame was updated to the (i−1)th frame. Finally, $u_{i}$ was estimated using (11) with the updated reference frame.

**Figure 8:**
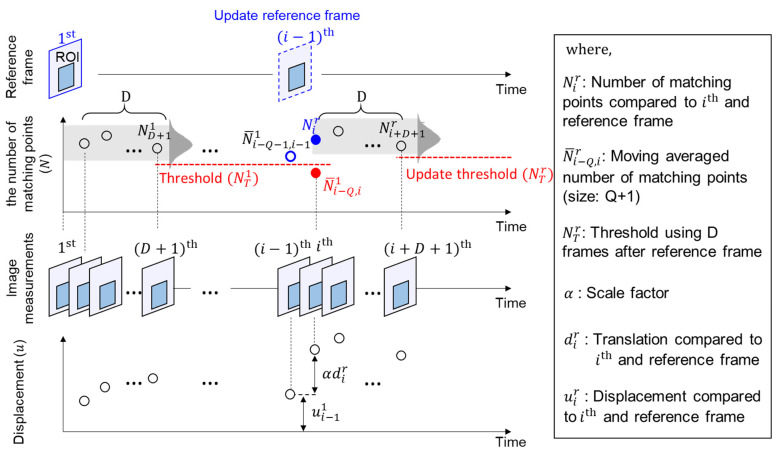
Example of reference frame and threshold updating process.

Figure 8 shows an example of the threshold updating process. The threshold value ($N_{T}^{1}$) was initially determined using D frames after the first frame. In the ith frame, the moving-averaged feature-matching number (${\bar{N}}_{i-Q,i}^{1}$) is less than $N_{T}^{1}$. Therefore, the reference frame is updated to the (i−1)th frame and the threshold is updated ($N_{T}^{r}$) using D frames after the (i−1)th frame.

The proposed algorithm was applied separately to vision and IR images. A vision camera was used during the day and an IR camera was used during the night. However, vision and IR images were acquired simultaneously during the transition time (e.g., day-to-night or night-to-day) and the numbers of matches were calculated respectively. Note that the transition time can be set to approximately 1 h before and after the beginning of the morning nautical twilight (BMNT) and the end of evening nautical twilight (EENT). Therefore, at the transition time, the robust number of features matching from the vision and IR images can be calculated as ${\bar{N}}_{V}$ and ${\bar{N}}_{I}$, respectively, and then compared. Displacement was estimated by selecting an application (vision or IR) with a large matching number.

## 3. Experimental Validation

To validate the performance of the proposed technique, a laboratory-scale test was conducted on a single-story building model with various excitation signals considering illumination and temperature variations, as described in Section 3.1, and a field experiment was conducted on a pedestrian bridge. Note that long-term measurement was impossible for the pedestrian bridge owing to safety issues, and it was difficult to consider the illumination and temperature variations in the field test. Therefore, only the applicability of the IR camera for displacement estimation under field conditions was verified in Section 3.2 by estimating the bridge displacement for a short period under stable illumination and temperature. In both tests, the displacement estimation performance of the proposed technique was compared to that of an existing technique [22] to highlight its superiority.

### 3.1. Lab-Scale Test Using Single-Story Building Model Test

**Figure 9:**
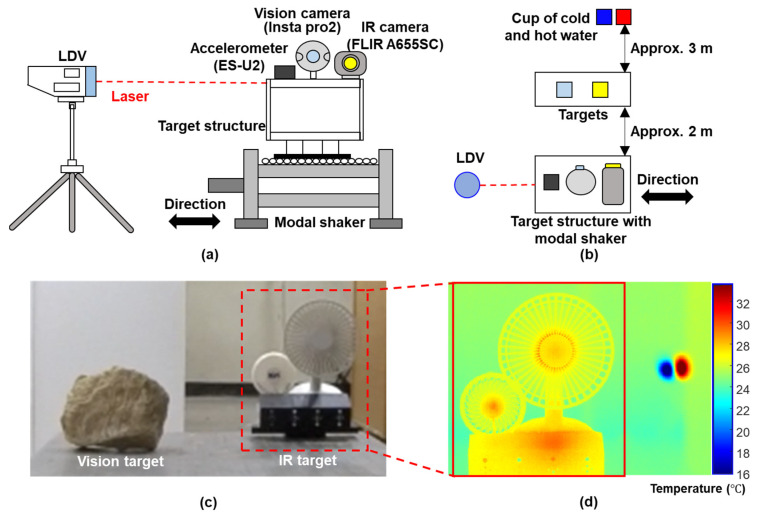
Overview of lab-scale test on a single-story building model: (**a**) front view of experiment setup, (**b**) top view of the experiment setup, (**c**) targets for vision and IR cameras, and (**d**) cropped IR image.

The proposed technique was first validated using a single-story building model test, the setup of which is illustrated in Figure 9a. A single-story building model composed of stainless steel was firmly attached onto an Electro-Seis APS 400 vibration shaker, which produced a horizontal movement of the building model. A Kinemetrix EpiSensor ES-U2 uniaxial force-balanced accelerometer, Insta360 Pro2 camera, and FLIR A655sc IR cameras were mounted on the top of the building model. A PSV-400-M4 LDV was used to measure the reference displacement of the building model with a resolution of 0.5 pm (Figure 9b). The sampling frequency for the vision and IR cameras was set to 30 Hz, and the acceleration and LDV measurements were sampled at 100 Hz. For ideal displacement estimation, a sufficient number of feature points should be detected within the ROI and the translations of all these features should be close to each other. In this study, a stone and two fans placed approximately 2 m from the building model were used as targets for the vision and IR cameras, respectively, as shown in Figure 9b,c. A cup of hot water and a cup of cold water were placed approximately 3 m away from the targets to simulate objects with high and low temperatures, and were included in the FOV of the IR camera, as shown in Figure 9d. Seven cases were considered to validate the proposed technique fully, as listed in Table 1. Note that the spatial resolutions of the vision and IR camera were 2880 by 3840 and 640 by 240, respectively, and all recorded images by the fisheye camera (insta360 pro 2) were calibrated by MATLAB built-in toolbox [26] for distortion correction.

#### 3.1.1. Initial Calibration Results (Case 1)

**Figure 10:**
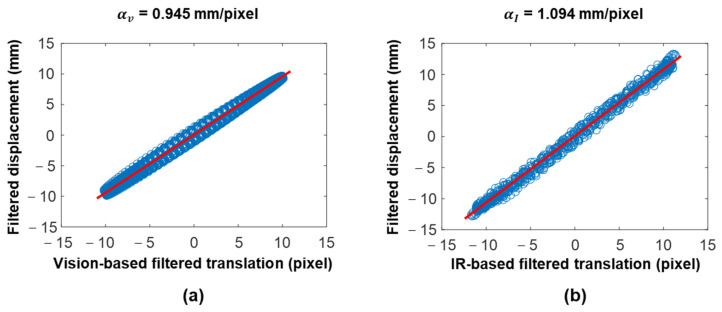
Scale factor estimations in the lab-scale test: (**a**) vision camera and (**b**) IR camera.

The scale factors for vision and IR measurements were estimated using the algorithm proposed in Section 2.1.1, exciting the building model with a 1 Hz sinusoidal signal. A bandpass filter was used before estimating the ratio between the acceleration-based displacement and the camera-based translation. The lower and upper cutoff frequencies of the bandpass filter were set to 0.3 Hz and 3 Hz, respectively, considering the effective frequency range of the accelerometer and the sampling rate of vision and IR cameras [24]. Figure 10 shows the estimated scale factors ($\alpha_{v}\;\mathrm{and}\;\alpha_{I}$) for the vision and IR cameras, which are 0.945 mm/pixel and 1.094 mm/pixel, respectively.

**Figure 11:**
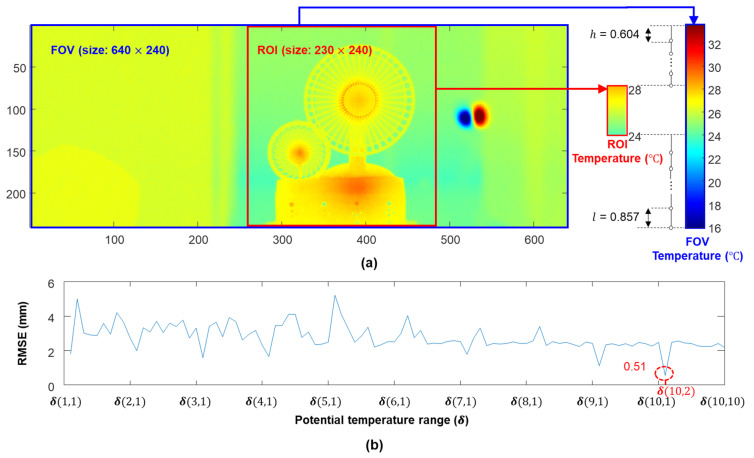
Temperature range optimization results: (**a**) temperature ranges of the ROI and field of view (FOV) and (**b**) RMSE under different temperature ranges.

Next, the temperature range was optimized for the IR measurements. Figure 11a shows the FOV of the IR camera and cropped ROI. The maximum and minimum temperatures were 33.52 °C and 15.94 °C, respectively, for the FOV due to the existence of two cups of water, while they were 28.08 °C and 23.65 °C, respectively, for the ROI. The differences between the maximum and minimum temperatures of FOV and ROI were equally divided into nine parts, and the values of l and h were set to 0.857 °C and 0.604 °C, respectively, as shown in a in Figure 11a. Potential temperature ranges were generated for different combinations of l and h, and the corresponding RMSEs were calculated, as shown in Figure 11b. Finally, the temperature range [16.79 °C, 28.08 °C] corresponding to the smallest RMSE was selected as the optimized temperature range.

#### 3.1.2. Displacement Estimation Results

(a) IR-based displacement estimation using optimized temperature range (Cases 2–5)

**Figure 12:**
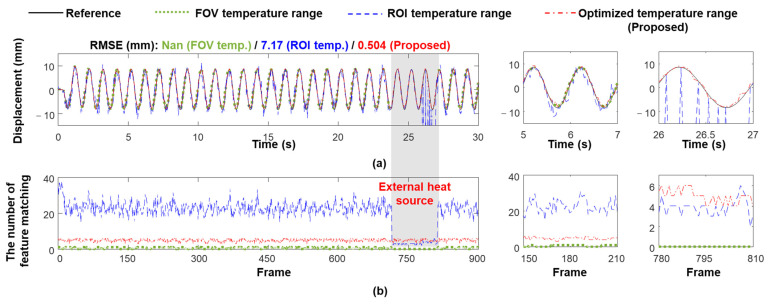
Comparison of (**a**) IR-based displacements and (**b**) number of matched feature points with an external heat source suddenly appearing in the ROI (Case 2).

The superiority of using the optimized temperature range was first verified when the building model was subjected to a 1 Hz sinusoidal signal excitation (Case 2). Note that the researcher’s finger appeared in the ROI at 23.5 s and disappeared from the ROI at 27 s to simulate an external heat source that may appear in the ROI in practice. Figure 12a compares the displacements estimated using the FOV and ROI temperature ranges, and the temperature range optimized by the proposed algorithm. The best displacement estimation performance was obtained using the optimized temperature range, indicating that the performance of IR-based displacement is sensitive to the temperature range. Note that when using the FOV temperature range, displacement cannot be continuously estimated and then its RMSE cannot be calculated. Figure 12b compares the number of matched feature points using the FOV, ROI, and optimized temperature ranges. Using the FOV temperature range generated many more matched feature points than using the ROI temperature range; however, the number of matched feature points decreased in both cases with the appearance of a finger (e.g., from 26 to 27 s). Therefore, during this period, the displacement was estimated with extremely large errors using the FOV temperature range, whereas the displacement could not be estimated using the ROI temperature range. However, the external heat source had less of an effect on the number of matched feature points when using the optimized temperature range, and sufficient feature points were stably matched to ensure a reliable displacement estimation.

**Figure 13:**
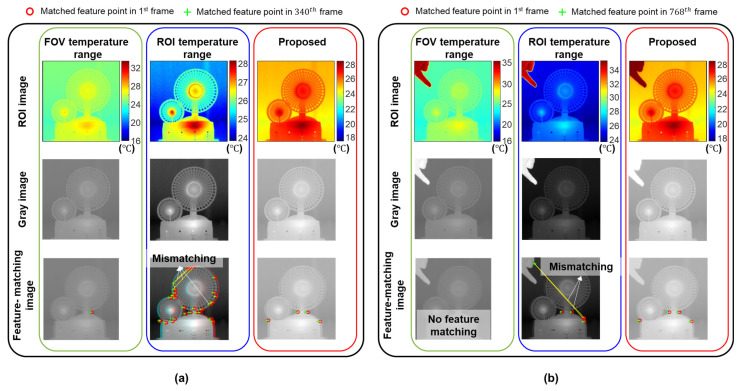
ROI, gray, and feature-matching images: (**a**) with and (**b**) without the external heat source (i.e., the finger).

Figure 13a compares the ROI images and corresponding matching results at the 340th frame without an external heat source (i.e., the finger). Only one feature point was matched when the FOV temperature range was used, because of the relatively small temperature variations within the ROI. Although a relatively large number of feature points (27) matched when the ROI temperature range was used, many were mismatched. Therefore, the displacement estimation accuracy was low in both cases. Figure 13b compares the ROI images and corresponding matching results at the 768th frame with the external heat source (i.e., the finger). No feature point was matched when the FOV temperature range was used, which caused failure in the continuous displacement estimation. The external heat source significantly reduced the number of matched feature points, and decreased the displacement estimation accuracy when using the ROI temperature range. However, the external heat source had less effect on the number of matched feature points when using the optimized temperature range, and four sufficient feature points were stably matched in both the 340th and 768th frames.

**Figure 14:**
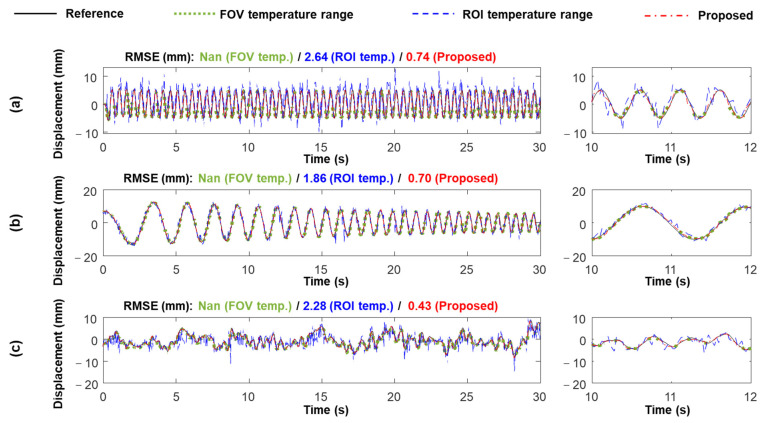
IR-based displacements estimated using FOV, ROI, and optimized temperature ranges under (**a**) 2 Hz sinusoidal (Case 3), (**b**) 0~3 Hz sweep (Case 4), and (**c**) recorded real bridge vibration signal (Case 5) inputs.

The superiority of using the optimized temperature range was further validated under three additional excitation signals, that is, a 2 Hz sinusoidal signal, 0–3 Hz sweep signal, and a recorded real bridge vibration signal. The displacements estimated using the different temperature ranges are compared in Figure 14. Using the optimized temperature range reduced the average RMSEs by 72.57% compared to using the ROI temperature range, and displacements were estimated accurately with RMSEs below 0.8 mm. Note that displacements were not continuously estimated using the FOV temperature range. Therefore, the RMSEs could not be calculated.

(b) Vision-based displacement estimation using adaptive reference frame updating (Case 6)

**Figure 15:**
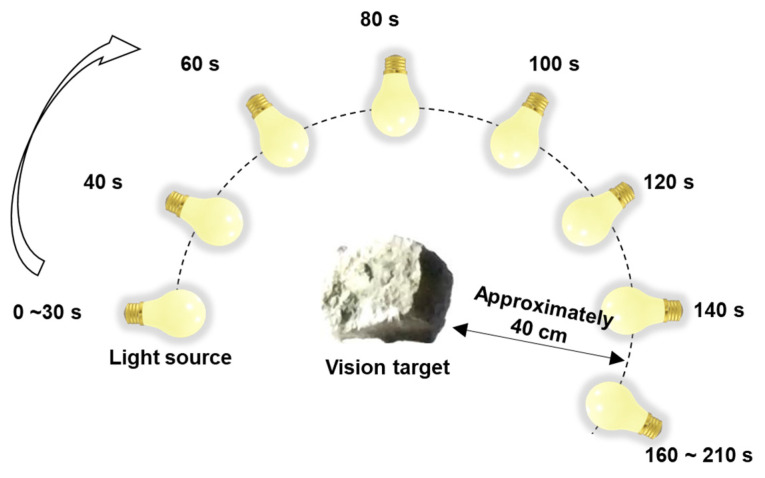
Simulation of varying illumination conditions for Case 6 of the lab-scale test using a moving light source (i.e., flashlight).

The superiority of the adaptive reference frame updating was verified under varying illumination conditions when the building model was simultaneously subjected to a 1 Hz sinusoidal signal and pseudo-static signal excitation (Case 6). A flashlight approximately 40 cm from the target of the vision camera was used as the light source and was moved, as shown in Figure 15, to simulate varying illumination conditions.

**Figure 16:**
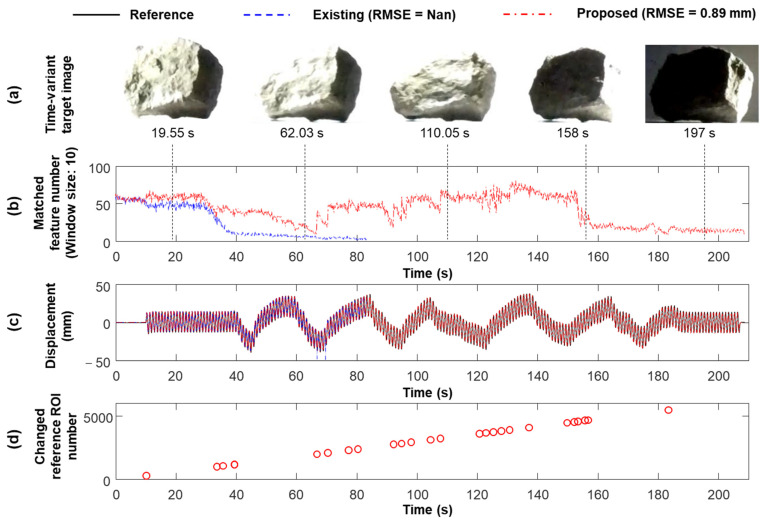
Estimation results Case 6 of the lab-scale test: (**a**) vision ROIs at different time steps, (**b**) vision-based displacements estimated with and without the proposed adaptive reference frame updating algorithm, (**c**) number of matched feature points with and without the proposed adaptive reference frame updating algorithm, and (**d**) time steps when updating the reference frame.

Figure 16a compares the captured target images at different times, and illumination variation is clearly observed. Figure 16b,c compare the number of matched feature points and estimated displacements using the existing and proposed techniques. The existing technique fixes the reference frame to the first frame, and enough feature points are matched in the first 30 s without illumination variations. Subsequently, the movement of the light source causes illumination variations in the ROIs. Therefore, the number of matched feature points decreased significantly until it reached zero at 80 s (Figure 16b), and the displacements were only estimated at 80 s (Figure 16c). However, the proposed technique adaptively updated the reference frame, and matched sufficient feature points under the dramatical illumination variation to continuously estimate displacement for 210 s with 0.89 mm RMSE (Figure 16b,c). However, the reference frame was updated 28 times using the proposed technique (Figure 16d), where dramatically varying illumination conditions were considered and the reference frame was updated frequently. However, considering slowly varying illumination under field conditions, less frequent frame updates are required.

(c) Continuous displacement estimation (Case 7).

**Figure 17:**
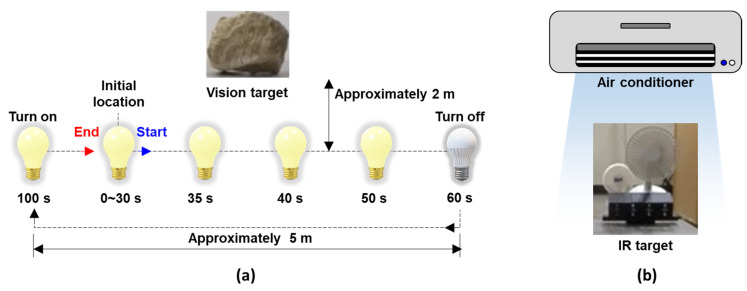
Simulation of (**a**) varying illumination and (**b**) varying temperature conditions in 24 h continuous displacement using the light source and air conditioner, respectively.

The proposed technique was validated by considering both illumination and temperature variations when the building model was subjected to a real bridge vibration signal excitation (Case 7). As shown in Figure 17a, to simulate the 24 h illumination variation in practice, the light source (i.e., the flashlight), approximately 2 m away from the target of the vision, slowly moved from the left side to the right side and then turned off at 60 s. Subsequently, it was moved to its original location and turned on for 100 s. Here, [0, 38 s], [38 s, 52 s], [52 s, 92 s], [92 s, 108 s], and [108 s, 120 s] simulate the day, the transition time from day to night, the night, the transition time from night to day, and during the day, respectively. When the light source was turned off, the air conditioner was turned on to simulate low-temperature conditions at night (Figure 17b).

**Figure 18:**
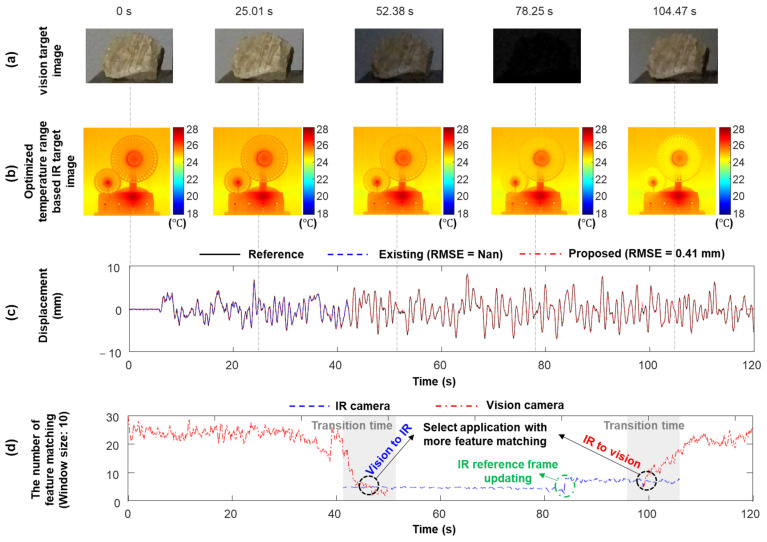
Estimation results for Case 9 of the lab-scale test: (**a**) vision ROIs at different time steps, (**b**) IR ROIs at different time steps using the optimized temperature range, (**c**) estimated displacements, and (**d**) the number of matched feature points.

As shown in Figure 18a, in the first 38 s, the illumination was relatively stable, and sufficient (more than 30) feature points were matched from the vision measurement. Therefore, the proposed technique estimates the displacement using vision and acceleration measurements. Subsequently, the illumination dramatically decreased, causing a decrease in the number of matched feature points in the vision measurement (Figure 18d). IR measurements were also obtained from the beginning of the transition from day to night (i.e., 41 s). Because the IR measurements were not sensitive to illumination variations, the number of matched feature points in the IR measurements was constant. When more feature points were matched from IR measurements rather than vision measurements (i.e., 45 s), the proposed technique switched to using IR and acceleration measurements to estimate displacement. At 82 s, the temperature variation induced by the air conditioner caused the ROI image of the IR measurement to be significantly different from that of the reference IR frame (i.e., the first IR frame), even when the optimized temperature range was used (Figure 18b). Therefore, the matched feature points were insufficient for displacement estimation, and the reference IR frame was updated to increase the number of matched feature points (Figure 18d). Vision measurements were obtained again from the beginning of the transition from night to day (92 s). When more feature points were matched from vision measurements than IR measurements (i.e., 100 s), the proposed technique switched back to using vision and acceleration measurements to estimate displacement. Through automated switching between the vision and IR cameras, the proposed technique continuously estimated the displacement with 0.41 mm RMSE (Figure 18c). However, displacements could only be estimated for the first 42 s using a vision camera and accelerometer.

### 3.2. Field Test

#### 3.2.1. Experimental Setup

**Figure 19:**
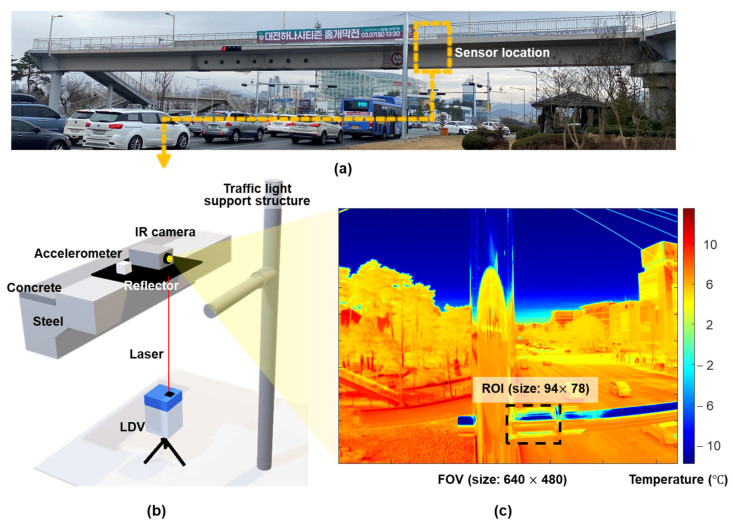
Overview of field test: (**a**) pedestrian steel box girder bridge, (**b**) sensor setup on bridge, and (**c**) view from the IR camera.

Figure 19 presents an overview of the field test on a pedestrian bridge. The bridge shown in Figure 19a is located in Daejeon, Korea, and has a length of 45 m and width of 8 m. An IR camera and uniaxial force balance accelerometer identical to those used in the lab-scale test were installed at approximately 1/4 of the span length of the bridge, as shown in Figure 19b. The main purpose of this test was to verify the applicability of the IR camera for displacement estimation under field conditions. A Polytec RSV-150 LDV was installed at a stationary location under the bridge to measure the reference displacement. Figure 19c shows the first frame of IR measurement. The pedestrian bridge was excited by four people jumping near the measurement point. Note that long-term measurement is required to consider temperature and illumination variation, but this is not possible owing to safety reasons. Therefore, the bridge displacements were estimated for a few minutes with stable illumination and temperature to verify the applicability of the IR camera for displacement estimation under field conditions.

#### 3.2.2. IR-Displacement Estimation Results

**Figure 20:**
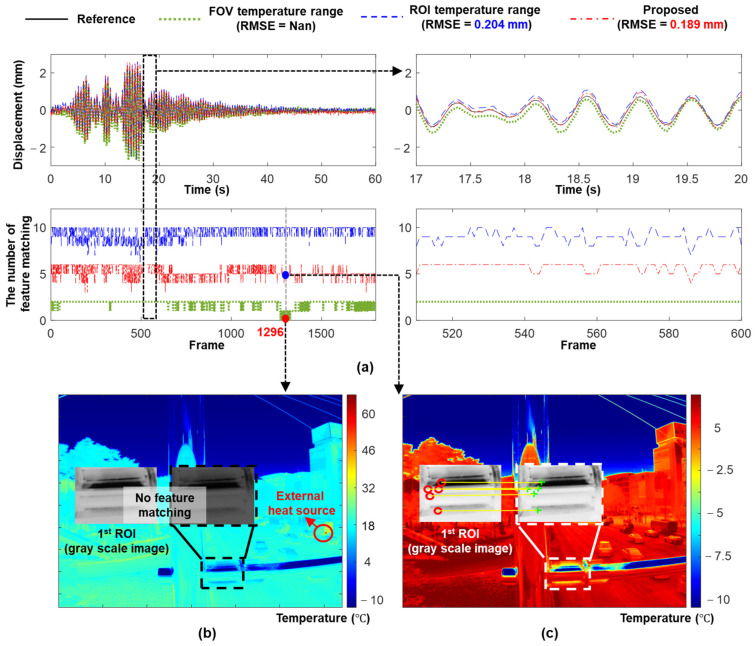
IR-based displacement estimation results: (**a**) IR-based displacement and matching results, (**b**) 1296th IR image (based on FOV temperature) before temperature optimization, and (**c**) after temperature optimization.

For the IR camera, a scale factor was estimated as 3.827 mm/pixel and the temperature range was optimized as [−10.2 °C, 6.4 °C]. The displacements estimated using the ROI, FOV, and optimized temperature ranges are compared in Figure 20a. The optimized temperature range exhibited the best displacement estimation performance with an RMSE of only 0.189 mm. Figure 20a shows a comparison of the number of matched feature points. Although the ROI temperature range had the most matched feature points, many of these feature points were mismatched and the displacement estimation accuracy was worse than that obtained using the optimized temperature range. Only a few feature points were matched when using the FOV temperature range, and a matching failure occurred when using an external heat source (e.g., bus exhaust). Therefore, the displacement could not be continuously estimated. However, the use of the optimized temperature ensured sufficient correctly matched feature points with and without a heat source (Figure 20b,c).

## 4. Conclusions

This study proposes a continuous structural displacement estimation technique using a collocated accelerometer, vision, and IR cameras. The proposed technique first estimates two scale factors for converting translation in a pixel unit to displacement in a length unit for the vision and IR cameras and then optimizes the temperature range for the IR camera. Subsequently, the displacement was continuously estimated by adaptively updating the reference frame and automated switching between the vision and IR cameras during the day and night. The main contributions of this study are (1) day and night continuous displacement estimation using an accelerometer, IR, and vision cameras; (2) automated temperature range optimization for the IR camera; and (3) adaptive reference frame updating for improved robustness against illumination and temperature variations. The proposed technique was validated through a laboratory-scale test that considered illumination and temperature variations, and the displacements were estimated using RMSEs below 1 mm. The applicability of the IR camera for displacement estimation under field conditions was validated using a pedestrian bridge test. The proposed reference frame updating improves the robustness against illumination and temperature variations but also causes error accumulation until the reference frame is updated back to the first frame. Further studies are required to address this issue. In addition, the proposed technique was validated only for a short time period, and further validation of its long-term performance under field conditions with illumination and temperature variations is required.

## Figures and Tables

**Table 1:** Descriptions of seven lab-scale test cases.

# of Cases	Test Duration (s)	Illumination Variation	Temperature Variation	Purposes
1	30	No	No	Initial calibration
2	30	No	Yes	Robustness of the proposed technique to external heat source
3	30	No	No	Performance of displacement estimation using optimized temperature range
4				
5				
6	210	Yes (Extreme)	No	Robustness of the proposed technique to extreme variations in illumination
7	120	Yes	Yes	Application selection of transition time for continuous displacement estimation

## Data Availability

Not applicable.
